# Validation of gene editing efficiency with CRISPR-Cas9 system directly in rat zygotes using electroporation mediated delivery and embryo culture

**DOI:** 10.1016/j.mex.2021.101419

**Published:** 2021-06-18

**Authors:** Anil K. Challa, Denise Stanford, Antonio Allen, Lawrence Rasmussen, Ferdinand K. Amanor, S. Vamsee Raju

**Affiliations:** a Department of Biology, University of Alabama at Birmingham, Birmingham, AL, USA; b Cystic Fibrosis Research Center, Division of Pulmonary, Allergy, and Critical Care Medicine, University of Alabama at Birmingham, Birmingham, AL, USA

**Keywords:** Chrna7, Indels, Homology-driven repair (HDR), Heteroduplex mobility assay (HMA)

## Abstract

Successful use of the CRISPR-Cas9 system for gene manipulation relies on identifying effective and efficient guide RNA sequences (gRNAs). When the goal is to create transgenic animal/rodent models by knocking-in desired sequences using homology-directed repair (HDR), selecting effective guides becomes even more critical to minimize developmental time and resources. Currently, validation experiments for gRNAs for generating rat models are carried out using immortalized rat cells. However, there are several limitations with using such cell lines, including ploidy of the genome, non-predictive transfection efficiency, and the ability to identify gene modifications efficiently within diverse cell populations. Since embryos are authentic representatives of live animals compared to cell lines, validating CRISPR guides for their nuclease activity in freshly isolated embryos will provide greater accuracy of *in vivo* gene editing efficiency. In contrast to microinjections, delivery by electroporation is a more accessible method that can be simple and does not require special skills and equipment. We demonstrate an accessible workflow to either delete or edit target genes *in vivo* in rats using the efficient editing of a human mutation in alpha7 nicotinic acetylcholine receptor subunit (*CHRNA7*) ortholog using electroporation as a delivery method for CRISPR-Cas9 ribonucleoprotein complexes in rat embryos.

• Upon identifying CRISPR targets at the desired genetic alteration site, we designed homologydriven repair (HDR) templates for effective and easy identification of gene editing by Restriction Fragment Length Polymorphism (RFLP).
• Cultured rat embryos can be genotyped to assess CRISPR activity as seen by either presence of indels resulting from NHEJ or knock-in of repair template resulting from homology driven repair.
• Heteroduplex mobility assay (HMA) and Restriction Fragment Length Polymorphism (RFLP) of PCR products can be performed reliably and reproducibly at a low-cost.

## Graphical abstract

**Figure:**
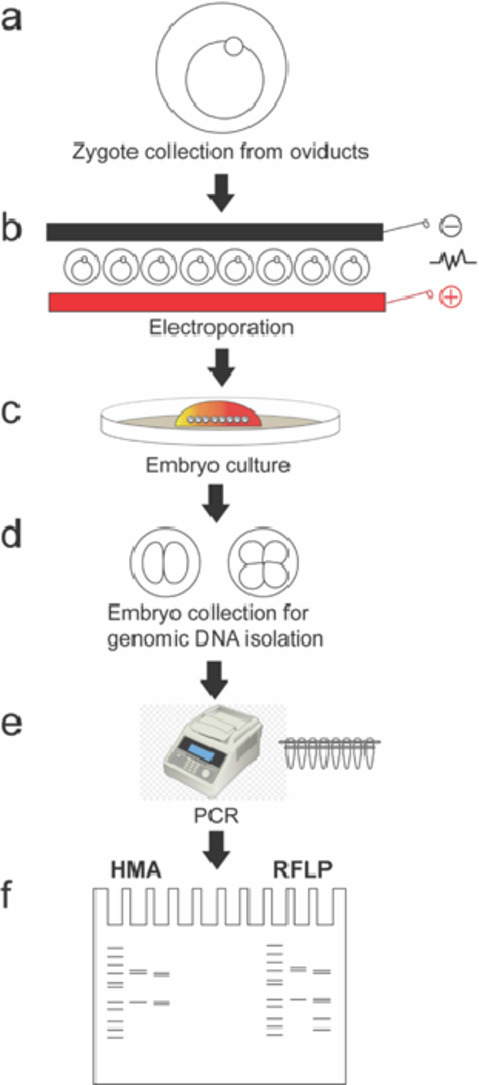

Specifications table

**Table:** 

Subject Area:	Biochemistry, Genetics and Molecular Biology Biochemistry, Genetics and Molecular Biology
More specific subject area:	*Genome Engineering*
Method name:	*CRISPR gene editing*
Name and reference of original method:	[1]
Resource availability:	Availability of all kits, plasmids, and other specialized materials are reported in the method details.

**Method details**

A. Bioinformatics analysis for design of CRISPR guides, repair template, and genotyping
  a. Tools
  b. Procedure
B. Guide RNA synthesis
  a. Materials and reagents
  b. Procedure
C. Repair template synthesis
D. In vitro nuclease assay
  a. Materials and reagents
  b. Procedure
E. Animal Use and embryo collection
  a. Materials and reagents
  b. Procedure
F. Electroporation and embryo culture
  a. Materials and reagents
  b. Procedure
G. Genotyping
  a. Materials and reagents
  b. Procedure
H. Method validation
  a. Summary of CRISPR editing in cultured embryos
  b. Summary of animal generation

## A. Bioinformatics analysis for design of CRISPR guides, repair template, and genotyping

a. Clustal Omega for multiple sequence analysis (https://www.ebi.ac.uk/Tools/msa/clustalo/)
b. Benchling for CRISPR design (https://benchling.com/)
c. IDT PrimerQuest for PCR primer design (https://www.idtdna.com/Primerquest/Home/Index)
d. Codon Usage regarding Restriction Finder (C.U.R.R.F.) for repair template design

*Procedure*:

To create animal models of specific human patient mutations in orthologous gene sequences, a thorough analysis of protein sequences must be performed to ensure that the position of the desired change is accurate. The exact positions (residue numbers) of specific amino acids might vary between species due to the presence of alternate transcripts, variations in exon lengths, and the number of total amino acids coded by the transcript. We performed multiple sequence analysis of human and rat protein sequences of the Chrna7 gene using Clustal Omega. While there are variations in the number of residues in the earlier part of the protein sequences (84% identities, 87% positives and 10% gaps), the alignment matches very well in the latter part of the sequence showing correspondence of the residue position to be edited (G423S).

After determining the exact position that needs to be edited in the rat gene sequence, CRISPR design tool in Benchling was used to identify potential guide sequences that can facilitate Cas9 nuclease activity at the desired site or in close proximity. This criterion generally limits the number of available guide sequences. In addition, guides that are predicted to have minimal off-target effects are preferred to increase specificity and reduce undesired double-strand breaks. We identified two guides – cut site of one of the guides was 11 bp away, and that of the other was 1 bp away from G423 of the rat ortholog of CHRNA7. Potential off-target sites are also provided, which can be help assess the specificity of the guides.

We designed PCR primers that would amplify a genomic fragment containing the CRISPR target site that is less than 1000 bp to analyze the nuclease activity and editing events at the target genomic locus reliably and efficiently. Other considerations for the PCR primers included 45-60% GC, 55-62°C annealing temperature, and 1 nucleotide 3’ GC clamp.

We used the Codon Usage regarding Restriction Finder (C.U.R.R.F.) tool to create the homologydriven repair template [2]. In addition to the amino acid change, we analyzed the codon degeneracy of the neighboring 6-10 residues (corresponding to 18-30 nucleotides), including the PAM site. To ensure that the modified allele that results from the CRISPR editing is not targeted by the guide RNA again, it is crucial to eliminate the PAM site (NGG) if possible. There are several instances when the PAM cannot be modified. In those instances, incorporating 2-3 silent mutations in the PAM proximal seed sequence can reduce the possibility of the guide RNA targeting the edited sequence [4]. In addition to this important aspect, creating a unique restriction enzyme site (preferably with a 6 bp recognition sequence) and these silent changes can be used as a proxy for the homology driven repair event. Restriction Fragment Length Polymorphism (RFLP) between the wildtype (unmodified) and edited (modified) sequences can be used to quickly identify a repair event during genotyping.

## B. Guide RNA synthesis

a. Oligonucleotide design and synthesis (www.benchling.com)
b. Generation of ds DNA template for in vitro guide RNA synthesis
c. In vitro transcription using T7 RNA Polymerase

### Materials and reagents

a. Forward oligonucleotide with T7 promoter, gene-specific target sequence, and ‘tail’ sequence
b. Reverse oligonucleotide with complementary ‘tail’ sequence
c. T4 DNA Polymerase
d. Ampliscribe T7 RNA synthesis kit
e. RNase-free DNase I
f. 5M Ammonium Acetate
g. 100% Ethanol
h. 70% Ethanol
i. RNA Storage Solution (Invitrogen, catalog# AM7001)

### Protocol

Gene specific oligonucleotide encoding with T7 RNA Polymerase promoter sequence (17 nt) upstream of gene target-specific sequence (20 nt) and a short stretch of the common guide RNA scaffold (15 nt) and the reverse complement of the common guide RNA scaffold sequence (80 nt) for *S.pyogenes* Cas9 are annealed at equimolar concentrations in 5 ul reaction volume. T4 DNA polymerase is used to create a dsDNA fragment with the partially annealed oligonucleotide pair in a 3’ filling reaction. The resulting dsDNA is used as a template in a T7 RNA polymerase-driven in vitro transcription reaction for 2 h. The template DNA is digested with an RNase-free DNase I enzyme followed by ammonium acetate and ethanol precipitation.

## Homology directed repair template synthesis

An asymmetric donor DNA as described by Richardson et al., (Richardson, Christopher D., et al. "Enhancing homology-directed genome editing by catalytically active and inactive CRISPR-Cas9 using asymmetric donor DNA." *Nature Biotechnology* 34.3 (2016): 339-344.) was designed and chemically synthesized long oligonucleotides Ultramers from IDT (4 nmol scale) were obtained.

## D. In vitro nuclease assay

### Materials and reagents

a. PCR product generated using gene specific primers and 2X Q5 Master Mix (NEB)
b. NEB Buffer 3.1
c. Alt-R® S.p. Cas9 Nuclease V3 (IDT, Catalog# 1081058)
d. RNAse A (100 mg/ml)
e. Proteinase K (20 mg/ml)
f. Acrylamide:bis-acrylamide (29:1) (Sigma)
g. 10% Ammonium Persulfate
h. TEMED (ThermoFisher, Catalog# 17919)
i. Tris base
j. Boric Acid
k. EDTA
l. SYBR Safe DNA staining dye
m. Gel imaging and documentation set up

### Procedure

1. Pre-assemble ribonucleoprotein complex of sgRNA (10–50 ng/mL) and Cas9 protein (10–50 ng/ul) with NEB Buffer 3.1 in a 10 ul reaction volume.
2. Add 1 ul of PCR amplified genomic fragment and mix by gentle finger vortexing.
3. Incubate for 30 minutes at 37 C
4. Add 0.5 ul of RNase A (100 ng/ul) to the reaction and incubate for 10 minutes at 37 C to digest excess sgRNA
5. Add 0.5 ul of Proteinase K (20 mg/ml) to the reaction and incubate for 10 minutes at 37 C to digest Cas9 protein. This step is essential since Cas9 binds to the template DNA and hinders its electrophoresis in a polyacrylamide gel.
6. Analyze the entire reaction volume (11 ul) in a 6% TBE-Polyacrylamide Gel Electrophoresis.
7. Stain the gel with DNA staining solution (1 ul SYBR Safe in 10 ml of nuclease-free water) by gently rocking for 5 minutes
8. Visualize and image the gel using UV transilluminator.

## E. Animal use and embryo collection

An approved animal protocol that allows maintenance of young adult rats, superovulation, and euthanasia should be in place to perform the experiments. The animal use described in this study was under APN 20157 and 20598 approved by the IACUC at the AAALAC accredited animal facility at University of Alabama at Birmingham. Since this study involves the optimization of a method based on previously published work, the ARRIVE guidelines were not comprehensively followed. However, all relevant standards in experiment design were meticulously followed.

### Materials and reagents

**Rats:** Sprague-Dawley rats were used in this study. Female rats at 5-6 weeks of age and male rats 812 weeks of age can be obtained from suppliers like Charles River and Taconic. Animals can also be locally procured if a home institution has an animal resource program that includes rats and may have excess young females that can be used as donors.

**Hormones:**

• Pregnant mare serum gonadotropin, PMSG (ProSpec, catalog# HOR-272-a)
• Human Chorionic Gonadotropin, hCG (ProSpec, catalog# HOR-250-a)
• Luteinizing Hormone Releasing Hormone, LHRH (ProSpec, catalog# HOR-261-a)

### Procedure for superovulation and collection of zygotes

Female rats between 3 and 5 weeks are injected with PMSG IP on day 1 and hCG IP on day 3 at 300 IU per kg body weight. Injections are done earlier in the day, and no later than 2 pm, along with a gap of 49.5 h between the first and second injections, has a positive effect on superovulation. All injections are done intraperitoneally. After the hCG injections, the females are paired with the male rats. On day 4, the donors are anesthetized by isofluorane followed by euthanasia by cervical dislocation to collect the oviducts carrying fertilized eggs.

In case female rats older than 7 weeks are utilized for superovulation, the estrous can be reset by injecting LHRH SubQ at a dose of 200 IU per animal and waiting for 48 h for the PMSF injection.

### Euthanasia and collection of oviducts

Superovulated female rats were euthanized with the induction of 5% inhaled Isoflurane for 10 minutes followed by cervical dislocation. Ovaries and oviducts were surgically exposed by making a 3 mm incision on the dorsal side 2 mm below last rib, directly over the ovary. The incision is made through the back muscle, and the ovarian fat pad was removed. The ovary and oviduct were exposed, and the oviduct was grasped using Dumont (#7) forceps to immobilize and cut away from the ovary using extra fine Bonn micro scissors [3]. Zygotes are released from the oviduct into prewarmed M2 media and moved to a drop of hyaluronidase (0.3 mg/mL) until the cumulus cells are dislodged from the zygotes. The clear zygotes are washed by moving sequentially into fresh 100-µl drops of prewarmed M2 media to remove any residual hyaluronidase. The zygotes are then pipetted into 10 µl drops of Opti-MEM media for equilibration.

## F. Electroporation and embryo culture

### Materials and reagents

a. EmbryoMax M2 medium, Millipore
b. Hyaluronidase, Millipore
c. KSOM/BSA medium, Millipore
d. mR1ECM media, CosmoBio
e. OptiMEM, Gibco
f. ECM830 Square Wave Electroporation System (BTX, Harvard Apparatus)
g. Oocyte slide electrode (BTX, 45-0496)

### Procedure

a. The final concentration of sgRNA was 50–100 ng/ul, that of Cas9 protein was 100–200 ng/ul, and the HDR template was 100 ng/ul. The electroporation solution can also include EGFP mRNA (50 ng/ml) as a reporter to assess the fidelity of the reagents and viability of developing embryos.
b. The sgRNA-Cas9 ribonucleoprotein complex is assembled in 10 µl Opti-MEM is mixed with the zygotes and deposited into a 1 mm gap of an oocyte slide electrode (BTX, 45-0496).
c. The electrode is connected to an ECM830 Square Wave Electroporation System (BTX, Harvard Apparatus) and treated to two pulses at 30 V for a pulse duration of 1 ms and pulse interval of 100 ms.
d. Immediately following the electroporation, the zygotes are transferred to 100 µl of prewarmed KSOM/BSA media, followed by washing them thrice in prewarmed mR1ECM media drops.
e. The zygotes are then transferred to prewarmed mR1ECM drops in a 60 mm petri dish and covered with embryo-grade mineral oil.
f. The petri dish is incubated under 5% CO_2_ and observed 24 hours later.

## G. Collection of 2-4 cell stage embryos and genotyping by PCR and RFLP

### Materials and reagents

Gene specific oligonucleotides (primers)

2x Q5 Polymerase Master Mix

XhoI restriction endonuclease

10X NEB CutSmart Buffer

6% TBE-polyacrylamide gel

Embryo lysis buffer

10mM Tris-HCL pH8

1mM EDTA

0.3% Tween 20

0.3% TritonX-100

### Procedure

Embryos are collected using a mouth pipette and placed individually or in groups (2–3) in 200 mL PCR tubes. After removal of any excess media, embryos can be directly mixed with 2x Q5 PCR master mix for amplification. When groups of embryos are analyzed as a batch, the embryos are lysed in embryo lysis buffer with Proteinase K (1mg/ml) at 55°C for 1 h. The Proteinase K is inactivated by incubation at 98°C for 10 min. The products obtained from the first round of PCR are analyzed by PAGE. In case of low yields of the desired amplicons, the PCR product can be treated with Exonuclease I and Shrimp Alkaline Phosphatase (Exo-SAP), diluted (1:50), and used as a template in the second round of PCR. The presence of higher bands indicates indels at the targeted site due to non-homologous end joining (NHEJ). Upon visualizing the amplicons, 1 uL of the PCR product is digested with the restriction endonuclease (XhoI) and NEB CutSmart buffer in a 10 L reaction volume at 37 C for 30 minutes. The digestion products are analyzed by 6% TBE-PAGE to detect the modified sequence resulting from homology directed repair (HDR). Since the wildtype sequence does not have the restriction site, an RFLP is observed only in the HDR modified fragment.

## H. Method validation

**Fig. 1:**
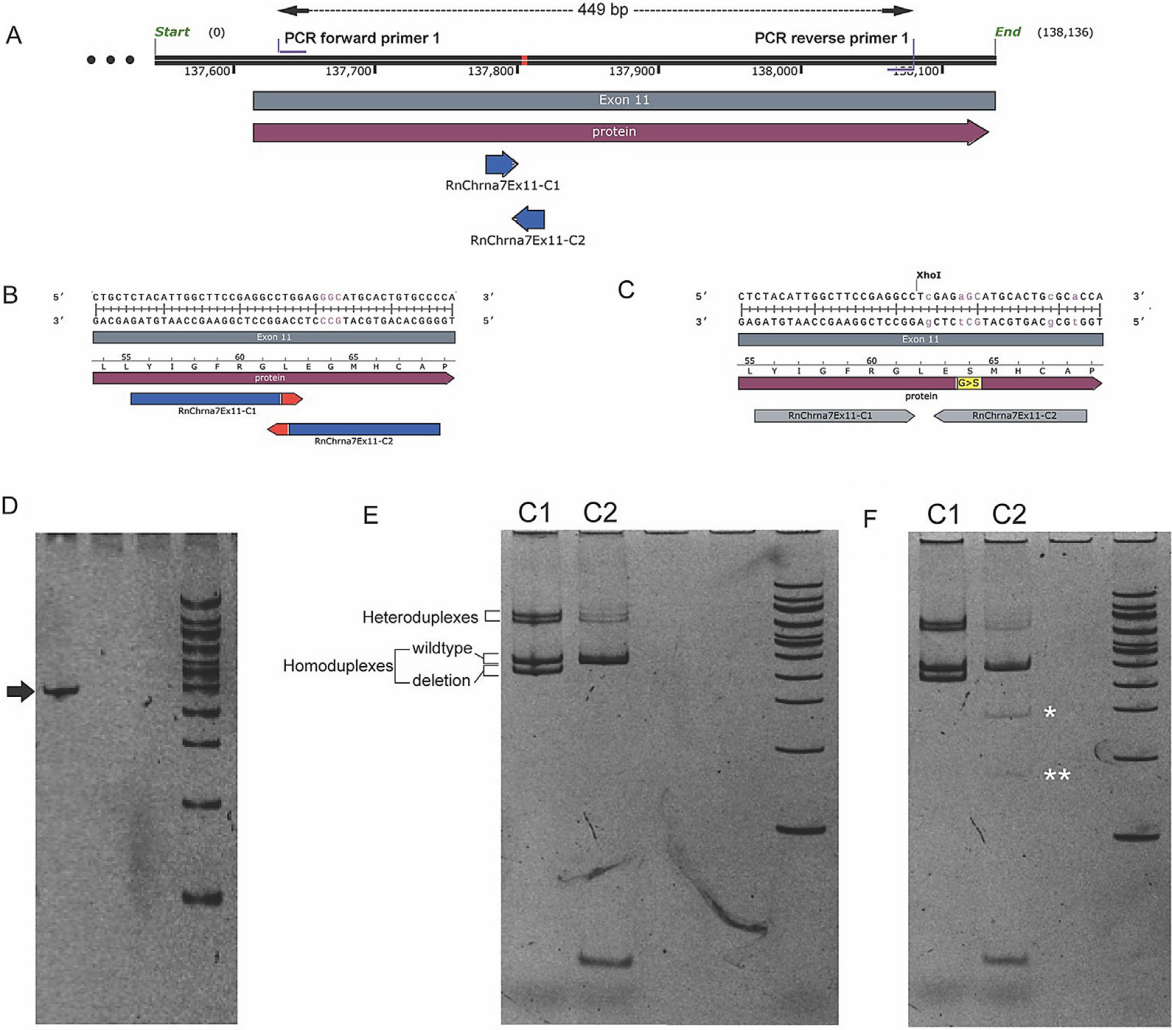
Assessing nuclease activity of sgRNA-Cas9 RNP complex in electroporated rat embryos. (A) Schematic of the genomic location along with CRISPR targets (blue arrows C1, C2) and PCR primers (F1, R1). (B, C) Schematics of the sequence at the exact target sites in the context of the wildtype (B) and modified (C) alleles. (D-F) Inverted images from 6% TBE-PAGE analysis of (D) PCR product of the wildtype allele. (E) HMA indicating the presence of heteroduplexes in PCR products obtained from zygotes electroporated with either guide C1 or C2. (F) RFLP analysis of the same PCR product with XhoI shows digested products indicated by white asterisks.

To create a missense/point mutation that results in the G423S change in the Chrna7 gene, we designed two sgRNAs in exon 11 to test nuclease activity and the ensuing HDR. The guides were in close proximity to the desired site in Exon 11 of Chrna7 – guide 1 (+) 5’-CTACATTGGCTTCCGAGGCC (TGG)-3’ and guide 2 (-) 5’-GGGCACAGTGCATGCCCTCC (AGG)-3’ (Fig. 1A). In vitro nuclease assay showed that the sgRNA-Cas9 ribonucleoprotein (RNP) complex was active (data are not shown).

To test the nuclease activity and HDR efficiency *in vivo*, we performed HMA and RFLP analysis using PCR products of primers F1: 5’-GAACTGGTGTGCATGGTTTC-3’ and R1: 5’-TGCCGATGGTACAGATGATG-3’. The analysis showed that both guides caused double-strand breaks leading to indel formation as seen by the presence of heteroduplexes (Fig. 1E). Also, HDR was seen in zygotes electroporated with guide C2 as can be inferred by the XhoI digestion products (Fig. 1F).

Further to the validation in cultured embryos, a litter of 16 rats was obtained after microinjecting 120 zygotes with the RNP complexes and transferring 96 healthy zygotes into 5 recipient rats. Three of the 16 pups (18%) had the desired point mutation, confirmed by Sanger sequencing. The modified allele with the desired point mutation was successfully transmitted through the germline into F1 generation.

## Declaration of Competing Interest

The Authors confirm that there are no conflicts of interest.
